# CMSP exerts anti-tumor effects on small cell lung cancer cells by inducing mitochondrial dysfunction and ferroptosis

**DOI:** 10.1515/med-2024-1100

**Published:** 2025-01-15

**Authors:** Xi Yan, Yinghao Niu, Yaojie Wang, Sisi Wei, Lina Han, Zhongyu Guo, Lianmei Zhao, Feng Gao

**Affiliations:** Department of Clinical Laboratory, The Fourth Hospital of Hebei Medical University, Shijiazhuang, 050011, China; Department of Clinical Biobank, The First Hospital of Hebei Medical University, Shijiazhuang, 050031, China; Research Center, The Fourth Hospital of Hebei Medical University, Shijiazhuang, 050011, China; Department of Thoracic Surgery, The Fourth Hospital of Hebei Medical University, Shijiazhuang, 050011, China

**Keywords:** *p*-hydroxyl cinnamaldehyde, CMSP, SCLC, mitochondrial dysfunction, ferroptosis, HMOX1

## Abstract

**Purpose:**

This study aims to investigate the role and mechanism of *p*-hydroxyl cinnamaldehyde (CMSP) in triggering ferroptosis of small cell lung cancer (SCLC) cells.

**Methods:**

The impact of CMSP on ferroptosis in H1688 and SW1271 cells was assessed through cell experiments and biological information analysis. Moreover, the expression of heme oxygenase 1 (HMOX1) in SCLC tissue was examined.

**Results:**

Following CMSP treatment, a concentration-dependent increase in cell death was observed, and differentially expressed genes were found to be associated with ferroptosis. CMSP notably facilitated ferroptosis events, such as elevated levels of reactive oxygen species (ROS), Fe^2+^, malondialdehyde (MDA), transferrin receptor 1 (TFR1), divalent metal transporter 1 (DMT1), and decreased levels of glutathione (GSH), solute carrier family 7 member 11 (SLC7A11), and glutathione peroxidase 4 (GPX4). Furthermore, CMSP promoted mitochondrial dysfunction, manifested as reduced mitochondrial volume, increased membrane density, elevated mitochondrial ROS, and decreased mitochondrial membrane potential. Consistently, the mitochondrial-targeted antioxidant Mito-TEMPO reversed CMSP-induced ferroptosis. Expression of the HMOX1 gene was markedly increased under CMSP treatment, while lower expression was observed in cancer tissue compared to adjacent tissue.

**Conclusion:**

CMSP triggers mitochondrial dysfunction via HMOX1 activation, leading to ferroptosis in SCLC cells, underscoring its potential as a therapeutic agent for SCLC.

## 1 Introduction

Small cell lung cancer (SCLC) is a subtype of lung cancer classified as a neuroendocrine tumor, representing around 15% of total lung cancer cases. It is known for its significant heterogeneity, high invasion, tendency for distant metastasis, and dismal prognosis [1]. SCLC is commonly categorized into limited-stage (LS) and extensive-stage (ES) according to the degree of spread, with around two-thirds of patients initially present with ES. While the utilization of effective chemotherapy agents and multidisciplinary treatment approaches has offered promise for certain individuals, progress in SCLC research has been sluggish, and the survival advantages for patients undergoing such comprehensive treatment regimens remain inadequate, culminating in overall poor survival rates [2]. Addressing urgent therapeutic challenges includes extending disease remission durations, circumventing tumor drug resistance, and tackling the predicament of limited options for subsequent-line treatments. Consequently, there is a critical necessity to explore novel therapeutic medications and targets to enhance the overall standard of care for SCLC.

Ferroptosis, as a novel form of cell death, differs from apoptosis, autophagy, and necrosis and is characterized by its reliance on iron ions [3]. Studies have shown that ferroptosis is closely related to the development of various tumors, such as colorectal cancer [4], breast cancer [5], and gastric cancer [6], and serves as a pivotal mechanism for inhibiting tumor growth. Additionally, key proteins in the related signaling pathways of ferroptosis can also serve as drug targets [7]. Furthermore, ferroptosis is also associated with tumor drug resistance. Cheng et al. [8] have demonstrated the advantages of targeting ferroptosis to overcome drug resistance in colorectal cancer, while Zhang et al. [9] have outlined the potential of ferroptosis as a new method to reverse cancer drug resistance. In recent years, ferroptosis has also attracted increasing attention in the treatment of lung cancer. Studies have confirmed that approved drugs such as metformin [10], Orlistat [11], and Erianin [12], as well as traditional Chinese medicine, extracts like Juglone [13] and Andrographolide [14], can impede the proliferation and invasion of lung cancer cells by inducing ferroptosis, providing new avenues for exploring alternative clinical treatments for lung cancer.

The cochinchina momordica seed (CMS) is a dried and ripe seed of *Momordica cochinchinensis* (Lour.) Spreng. (Family: Cucurbitaceae), primarily cultivated in southern China. This traditional Chinese medicine is often used in conjunction with other medications for the treatment of various tumors. *p*-Hydroxyl cinnamaldehyde (CMSP) is an important component of ethanol extract from CMSs. Initially discovered by our research team, it was confirmed to possess anti-tumor properties against melanoma cells and esophageal cancer cells [15,16,17]. However, the effects of CMSP on SCLC and its underlying mechanisms remain elusive. In this study, we identified ferroptosis enrichment through bioinformatics analysis in CMSP treatment with SCLC cells, validated the effect of CMSP on ferroptosis in two SCLC cell lines, and primarily explored the mechanism of CMSP-induced ferroptosis from the perspective of mitochondrial function and highlight on the essential role of CMSP in treatment of SCLC patients.

## 2 Materials and methods

### 2.1 Cell culture

The SCLC cell line H1688 was obtained from the Cell Bank of the Institute of Biochemistry and Cell Biology at the Chinese Academy of Sciences (Shanghai, China), and SW1271 was sourced from Meisen Cell Technology Co., Ltd (Zhejiang, China). H1688 was cultured in RPMI1640 medium (Gibco, USA) with 10% heat-inactivated fetal bovine serum (BI, USA), and SW1271 was cultured in DMEM (Gibco, USA) with the same serum concentration. The cell cultures were maintained at 37°C in a 5% CO_2_ environment.

The CMSP group involved the cells cultured in a medium with varying concentrations of CMSP for 24 or 48 h. The CMSP + ferrostatin-1 group involved an initial 12-h incubation in a medium containing 2 μmol/L ferrostatin-1 (MedChemExpress, USA), followed by a change to a medium containing CMSP (20 μg/ml) for an additional 48 h. The CMSP + Mito-TEMPO group received CMSP (20 μg/ml) and Mito-TEMPO (1 μM, Aladdin, China) for a 48-h incubation. The solvent control group was cultured for 48 h with an equivalent amount of dimethyl sulfoxide (DMSO).

### 2.2 Extraction and isolation of CMSP

CMSP is an ethanol extract derived from the Chinese herbal medicine *Momordica cochinchinensis* and manufactured by Fugu Medical Technology Company (Wuxi, China). The detailed synthetic procedure and chemical composition of CMSP are based on our earlier research [16], and the resulting CMSP is a light yellow solid (the purity is 99.9%). CMSP is dissolved in dimethyl sulfoxide for storage (100 mg/ml) and diluted with serum-free culture medium before use, ensuring a final DMSO concentration of <0.01%. The specific structure of CMSP is as depicted in previous research.

### 2.3 Observation of cell morphology and cell viability assay

The cells were seeded at a density of 3 × 10^3^ cells per well in a 96-well plate. Following the treatment, alterations in cell morphology were investigated using an inverted phase contrast microscope (Olympus, Japan). Cell viability was determined by quantifying the optical density (OD) at 490 nm using the MTS assay (Promega, China) to assess cell viability. The experiment was replicated three times.

### 2.4 Clone formation assay

In the logarithmic growth phase, H1688 and SW1271 cells were seeded at a concentration of 2 × 10^3^ cells per well in a 6-well plate. Various concentrations of CMSP were introduced for ongoing cultivation. The development of clones was monitored and quantified on the 10th day [17].

### 2.5 Cell death assays by flow cytometry

The CMSP-treated cells were analyzed using the Annexin V/7-AAD assay kit from BD (USA) to determine the proportion of dead cell populations (double positive for Annexin V and AAD).

### 2.6 mRNA sequencing and bioinformatics analysis

Total RNA was isolated from H1688 cells treated with CMSP (10 μg/ml). The integrity of RNA was evaluated using an Agilent Bioanalyzer 2100 (Agilent Technologies, Santa Clara, CA, USA), and the concentration and purity of total RNA were determined using the Qubit^®^ 3.0 Fluorometer (Life Technologies, CA, USA) and Nanodrop One spectrophotometer (Thermo Fisher Scientific Inc, USA). Subsequently, mRNA sequencing was conducted by Sinotech Genomics, China. The sequencing data were analyzed using the Hisat2, Samtools, and Htseq-count software packages. Gene expression analysis was performed with Cufflinks, and volcano plots and heat maps were generated using the R language. Additionally, pathway enrichment analysis of differentially expressed genes was carried out using the GSEA software package. The STRING database was used to construct the protein–protein interaction (PPI) network, and Cytoscape software (v3.9.1) was used to screen the hub genes.

### 2.7 Detection of reactive oxygen species (ROS)

Following the protocol of the assay kit, the treated cells from each group were incubated with the DCFH-DA (Nanjing jiancheng, China) probe at 37°C for 30 min. Subsequently, the cells were washed twice with PBS. Cell staining levels were observed under a fluorescence microscope, or the cells were collected for flow cytometry analysis.

### 2.8 Lipid peroxidation and Fe^2+^ assay

H1688 and SW1271 cells were grouped in accordance with the methodology described in Section 2.7. The concentrations of malondialdehyde (MDA; Nanjing jiancheng, China), glutathione (GSH; Nanjing jiancheng, China), and Fe^2+^ (Solarbio, China) were determined using the corresponding assay kits.

### 2.9 Western blot

As described in Section 2.7, proteins from each group were individually extracted, and their concentrations were measured using the BCA assay kit (Biosharp, China). The relative expression levels of the target protein were determined by following the procedure outlined in previous studies [17,18,19]. The primary antibodies utilized were anti-glutathione peroxidase 4 (GPX4; Proteintech, China), anti-solute carrier family 7 member 11 (SLC7A11; Proteintech, China), anti-divalent metal transporter 1 (DMT1; Proteintech, China), anti-transferrin receptor 1 (TFR1; Proteintech, China), and anti-GAPDH sourced from Proteintech in China.

### 2.10 Mito-ROS assays

Mitochondrial ROS levels were detected using the MitoSox Red probe (Invitrogen, USA) [20]. The treated cells were incubated with 5 μM of the MitoSox Red probe at 37°C for 30 min, and the fluorescence intensity was measured under a microscope (Olympus, Japan).

### 2.11 Mitochondrial membrane potential assays

The treated cells underwent testing to evaluate alterations in mitochondrial membrane potential in accordance with the guidelines of the JC-1 mitochondrial membrane potential assay kit (Nanjing jiancheng, China).

### 2.12 Immunohistochemistry

We obtained ten cases of surgically removed SCLC tissues and their corresponding adjacent normal tissues from our hospital during the period 2017–2023. The tissue sections, 4 mm in thickness, were processed, subjected to 1-h baking at 65°C, and then stained using the automated Roche immunohistochemistry staining system, following the standard protocol recommended by the manufacturer. The staining reagents used in this study included hematoxylin II, bluing reagent, and the Universal DAB Detection Kit for ultraviolet light. We used a mouse anti-human heme oxygenase 1 (HMOX1) monoclonal antibody (1:1,000, Proteintech, China) as the primary antibody, with diaminobenzidine (DAB) as the chromogen. Imaging was carried out using an imaging system from Japan. In adherence to the prior grading protocol [21], every histopathological slide was independently evaluated by two seasoned pathologists. Before surgery, none of the cases received any form of anti-tumor therapy.

### 2.13 Statistical analysis

Statistical analysis was conducted using SPSS 25.0 or GraphPad Prism 9.0. Descriptive statistics were applied to represent the experimental data using mean ± SD. Intergroup comparisons were performed using Student’s *t*-test and one-way analysis of variance. Statistical significance was defined as a *p*-value < 0.05.


**Ethical approval:** This study obtained approval from the Ethics Committee of the Fourth Hospital of Hebei Medical University (approval No. 2019057).
**Informed consent:** Informed consent was obtained from either the patients or their legal guardians.

## 3 Results

### 3.1 CMSP suppressed the proliferation of SCLC cells and induced cell death

**Figure 1:**
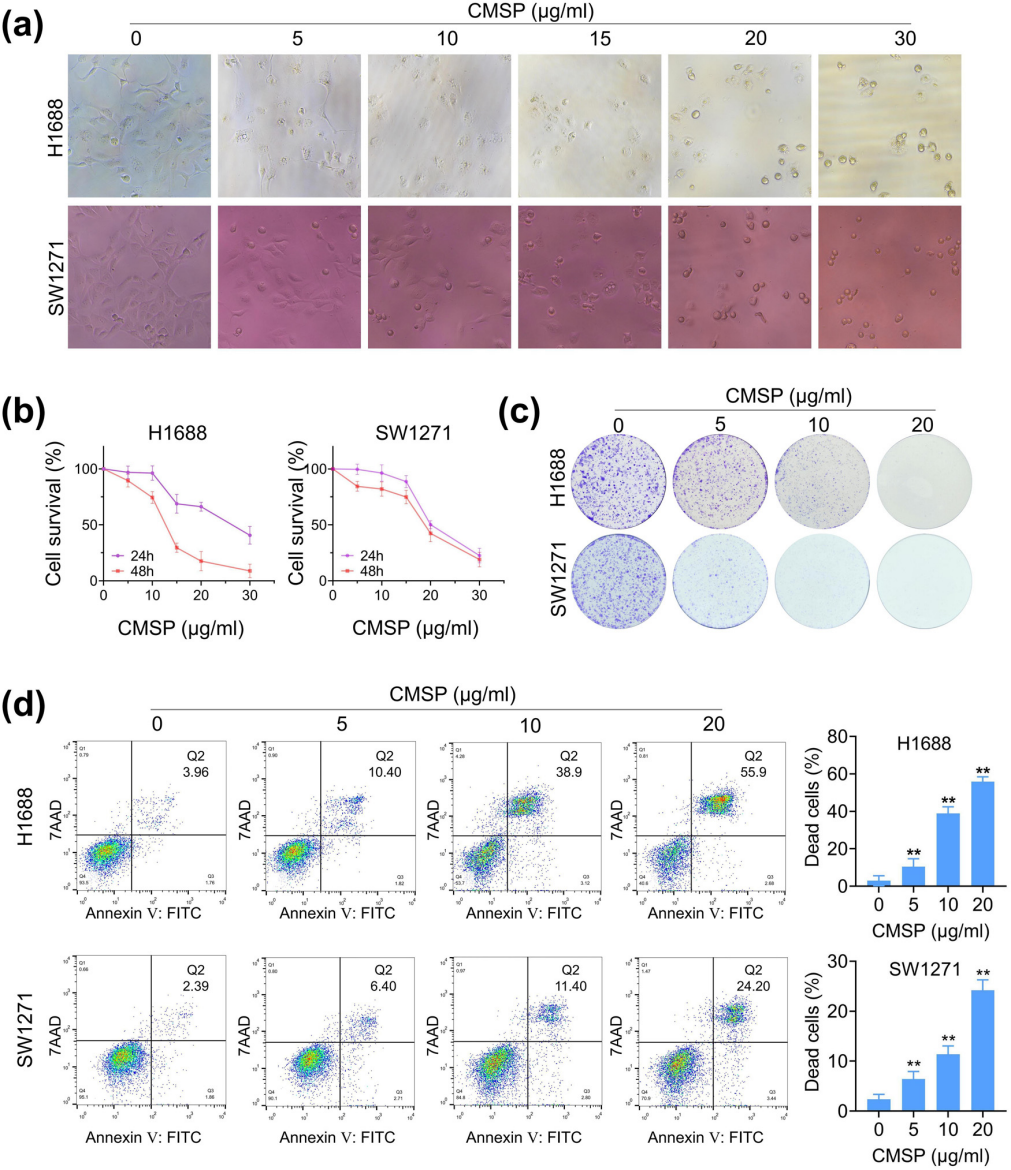
Effects of CMSP on the proliferation and death of SCLC cells. (a) Morphological changes in H1688 and SW1271 cells (200×) after treatment with different concentrations of CMSP for 48 h. (b) Effect of CMSP on the vitality of H1688 and SW1271 cells. (c) Effect of CMSP on the colony formation ability of H1688 and SW1271 cells. (d) Percentage of 7AAD-positive cells (dead cells) in H1688 and SW1271 cells detected by flow cytometry after CMSP treatment. Values are expressed as mean ± SD and were obtained from triplicate experiments. **P < 0.01, versus the control group.

To investigate the potential anti-tumor effects of CMSP on SCLC cells, H1688 and SW1271 cells were treated with various concentrations of CMSP (0, 5, 10, 15, 20, and 30 μg/ml) for 24 and 48 h. The effects of CMSP on the morphology, viability, proliferation, and death of the lung cancer cells were assessed. The H1688 and SW1271 cells without treatment exhibited large, full, smooth, and densely packed morphology with vigorous growth. Upon CMSP treatment, the cells displayed reduced volume, rounder shape, decreased cell count, and scattered growth (Figure 1a). Additionally, MTS assay results indicated a significant, concentration- and time-dependent reduction in cell viability following CMSP treatment (*P* < 0.05, Figure 1b). The IC50 values for CMSP at 24 h were 25.235 and 21.630 μg/ml, and at 48 h were 12.261 and 18.365 μg/ml for H1688 and SW1271 cells, respectively. Subsequent colony formation assays revealed marked suppression of colony formation in H1688 and SW1271 cells compared to the control group (Figure 1c). Moreover, flow cytometry analysis revealed a dose-dependent increase in the proportion of Annexin V+/7AAD+ dead cells with increasing CMSP concentration (*P* < 0.05, Figure 1d). This implies that CMSP has the potential role in impeding SCLC cell proliferation *in vitro* and inducing cell death in H1688 and SW1271 cells.

### 3.2 RNA-Seq analysis of CMSP-induced cell death in SCLC

**Figure 2:**
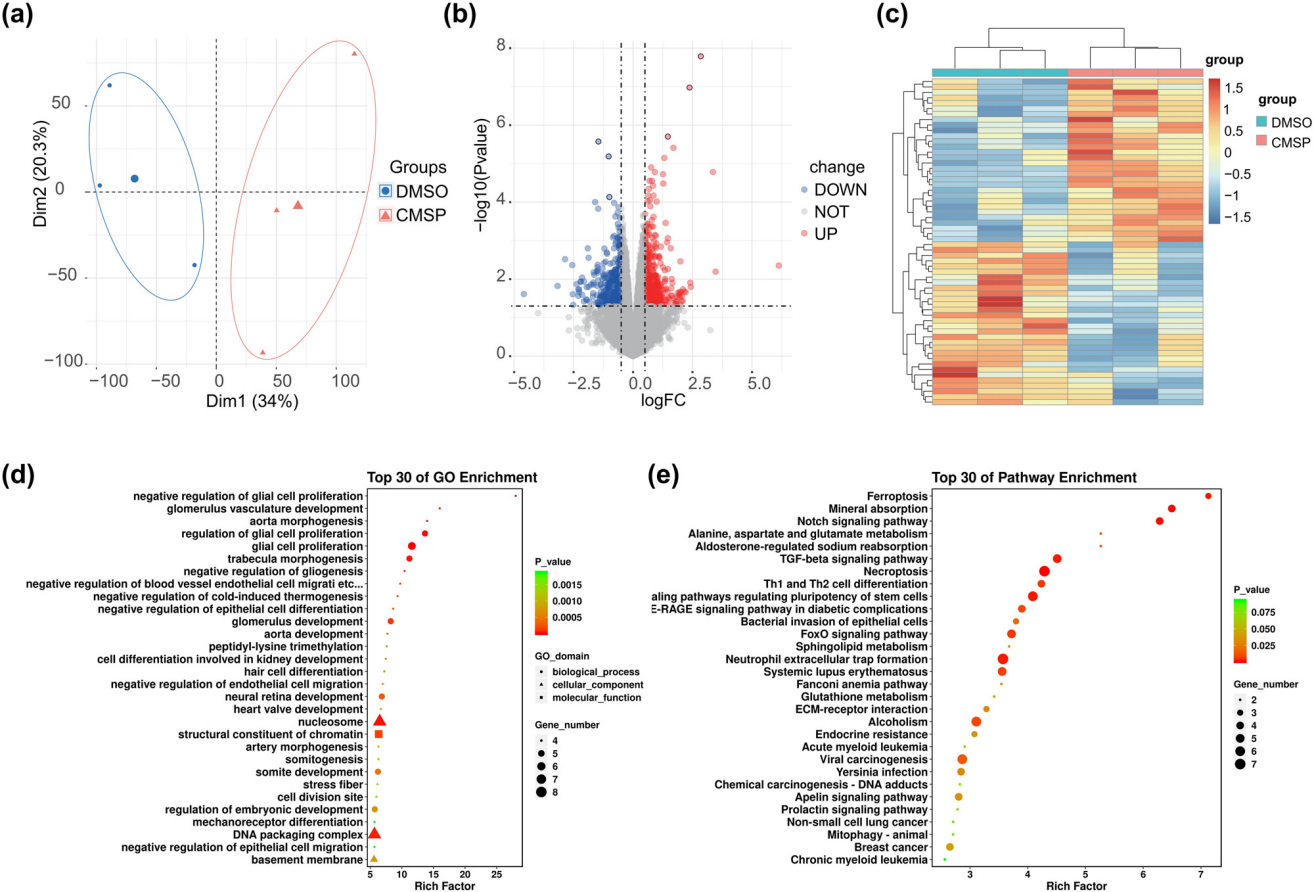
Differential gene analysis of H1688 cells in the CMSP-treated and the untreated groups. (a) Principal component analysis of the samples; (b) volcano plot of differential genes in H1688 cells of CMSP-treated and untreated groups; (c) heat map of differential genes in H1688 cells of CMSP-treated and untreated groups; (d) GO enrichment analysis of differential genes in H1688 cells of CMSP-treated and untreated groups; and (e) KEGG pathway analysis of differential genes in H1688 cells of CMSP-treated and untreated groups.

To elucidate the molecular mechanism of CMSP in induced SCLC cell death, RNA-Seq sequencing was conducted on CMSP-treated H1688 cells. Initially, gene expression data were standardized, and the principal component analysis demonstrated a consistent distribution of three replicates within each group (Figure 2a), a testament to the reliability of the data. Subsequent analysis indicated that, compared to the control group, the CMSP treatment group exhibited 379 upregulated genes (log2 fold change > 0.5; *P* < 0.05) and 375 downregulated genes (log2 fold change < −0.5; *P* < 0.05) (Figure 2b and c). Differential expression of genes (DEGs) prompted GO and KEGG signaling enrichment analyses. The GO analysis revealed notable enrichment of genes primarily involved in the regulation of cell proliferation (Figure 2d). Furthermore, the KEGG analysis showed significant enrichment of genes linked to pathways associated with ferroptosis, necroptosis, Th1 and Th2 cell differentiation, and various aspects of metabolism, with the ferroptosis pathway being notably enriched (as depicted in Figure 2e). These findings suggest that the ferroptosis signaling pathway may play a pivotal role in CMSP-induced SCLC cell death.

### 3.3 CMSP promotes ROS-induced ferroptosis in SCLC cells

**Figure 3:**
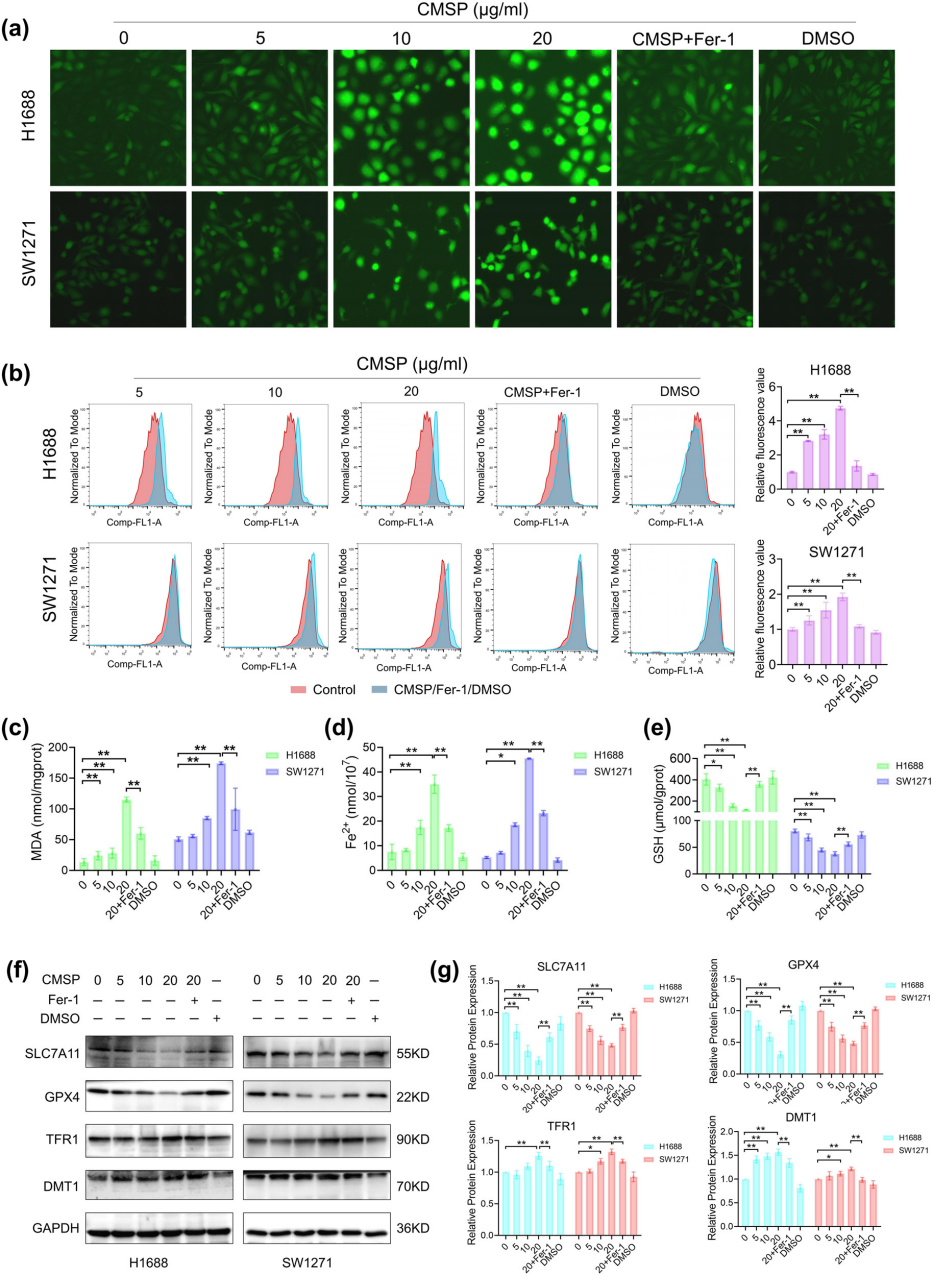
CMSP induces ferroptosis in SCLC cells. H1688 and SW1271 cells were treated with CMSP with or without Fer-1 (1 μM) for 48 h, (a) fluorescence microscopy images of DCFH-DA probe in cells; (b) assessment of ROS by flow cytometry using DCFH-DA probe fluorescence intensity; (c) detection of MDA levels using an MDA assay kit; (d) detection of Fe^2+^ levels using a Fe^2+^ assay kit. (e) detection of GSH levels using a GSH assay kit; (f) and (g) measurements of the expression levels of ferroptosis-related proteins SLC7A11, GPX4, TFR1, and DMT1 by western blotting. Values are expressed as mean ± SD and were obtained from triplicate experiments. *P < 0.05 and **P < 0.01.

To assess the potential of CMSP in inducing cell ferroptosis, we analyzed key biochemical markers, namely ROS, GSH, MDA, and Fe^2+^. After CMSP treatment, a gradual increase in the levels of cellular ROS (Figure 3a and b), MDA (Figure 3c), and Fe^2+^ (Figure 3d) was observed, while GSH levels (Figure 3e) declined. Furthermore, we evaluated the expression of pivotal proteins involved in ferroptosis, including GPX4, SLC7A11, TFR1, and DMT1. The findings revealed that CMSP suppressed the expression of GPX4 and SLC7A11 (*P* < 0.01) and induced the expression of TFR1 and DMT1 (*P* < 0.01) in a concentration-dependent manner, as illustrated in Figure 3f and g. Additionally, verification of CMSP’s induction of cell ferroptosis involved co-treatment with the ferroptosis-specific inhibitor, ferrostatin-1. The results demonstrate that ferrostatin-1 could counteract the effects of CMSP on the aforementioned parameters. In summary, these outcomes provide evidence that CMSP induces ferroptosis in H1688 and SW1271 cells.

### 3.4 CMSP-induced mitochondrial dysfunction in SCLC cells

**Figure 4:**
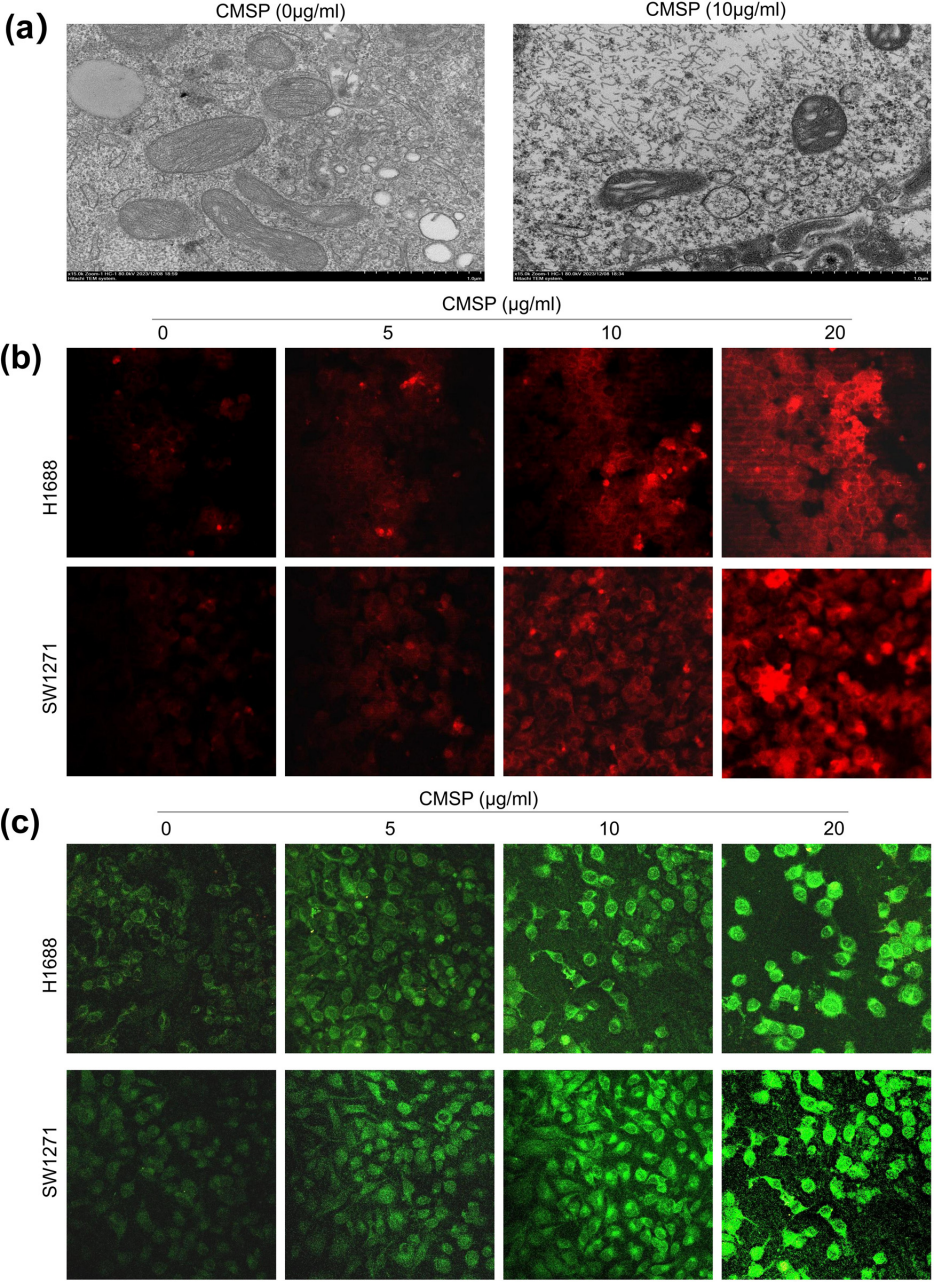
CMSP induces mitochondrial dysfunction. H1688 and SW1271 cells were treated with different concentrations of CMSP: (a) the observed changes in mitochondrial morphology using scanning electron microscopy; (b) detected mitochondrial ROS levels using the MitoSox Red probe; (c) assessed mitochondrial membrane potential using the JC-1 mitochondrial membrane potential assay kit. Values are expressed as mean ± SD and were obtained from triplicate experiments. **P < 0.01, versus the control group.

Mitochondrial dysfunction and heightened oxidative stress play crucial roles in the regulation of ferroptosis. To further investigate the impact of CMSP on mitochondrial function, we examined the ultrastructural changes in mitochondria using transmission electron microscopy. Figure 4a shows that treatment with CMSP (10 μg/ml) induced mitochondrial shrinkage, increased double membrane density, and reduced cristae in H1688 cells. The analysis of mitochondrial ROS levels using the MitoSox Red probe demonstrated a gradual elevation in mitochondrial superoxide levels in H1688 and SW1271 cells with increasing concentrations of CMSP (Figure 4b). Furthermore, JC-1 staining depicted a notable increase in JC-1 monomer green fluorescence after CMSP treatment in both cell lines, suggesting a decline in the mitochondrial membrane potential (Figure 4c). These findings suggest that CMSP triggers an elevation in mitochondrial ROS in a concentration-dependent manner while simultaneously diminishing the mitochondrial membrane potential, ultimately leading to mitochondrial impairment. The above results demonstrate that mitochondria-targeted antioxidants effectively mitigate CMSP-induced mitochondrial dysfunction, implying that CMSP triggers ferroptosis by inducing mitochondrial dysfunction in SCLC cells.

### 3.5 CMSP triggered mitochondrial dysfunction in SCLC cells leading to ferroptosis

**Figure 5:**
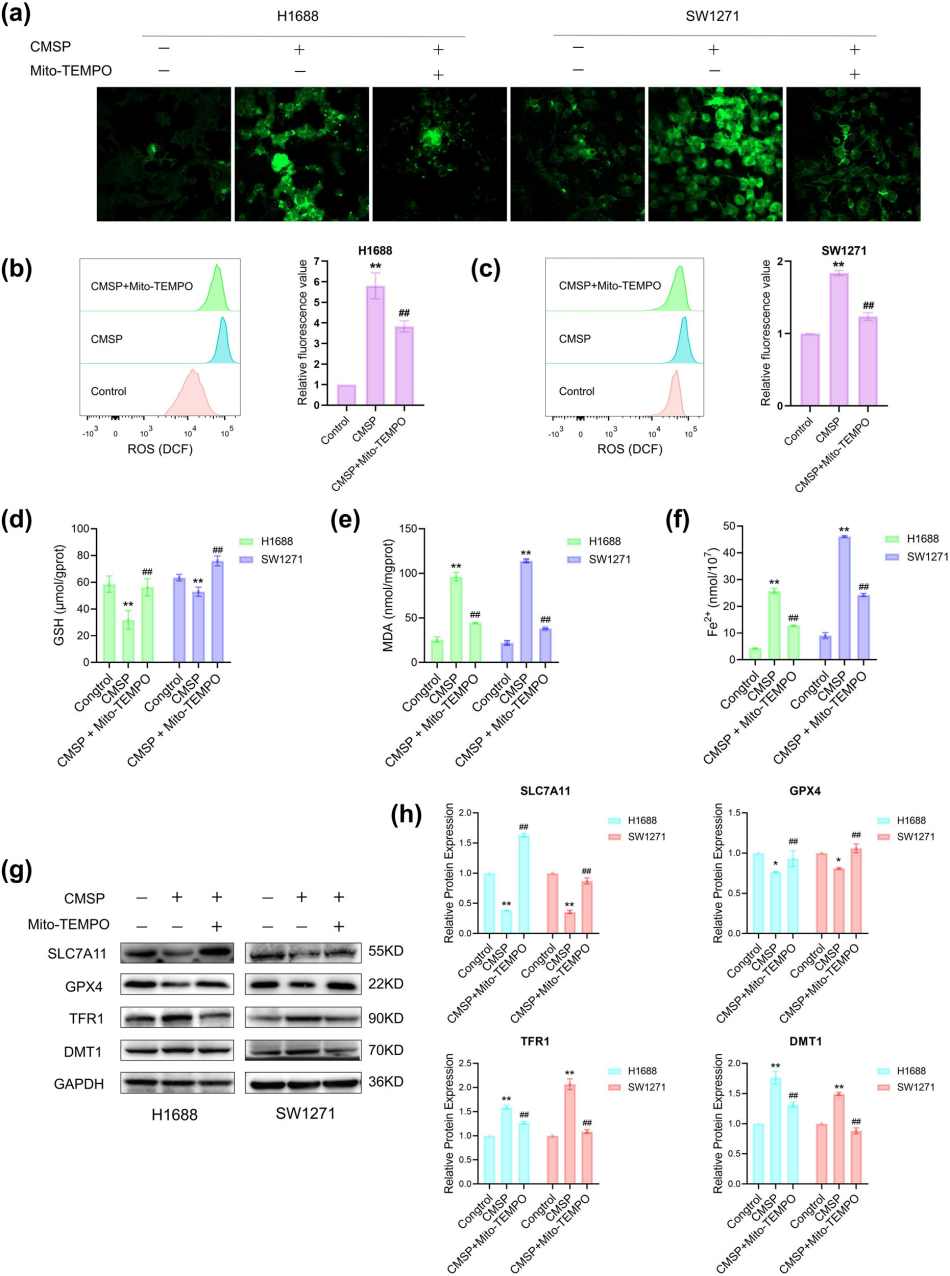
Ferroptosis induced by CMSP is related to mitochondrial dysfunction. H1688 and SW1271 cells were treated with CMSP (20 μg/ml) with or without Mito-TEMPO (1 μM) for 48 h: (a) fluorescence microscopy images of DCFH-DA probe in cells; (b) and (c) assessment of ROS by flow cytometry using DCFH-DA probe fluorescence intensity; (d) detection of GSH levels using a GSH assay kit; (e) detection of MDA levels using an MDA assay kit; (f) detection of Fe^2+^ levels using a Fe^2+^ assay kit; (g) and (h) measurements of the expression levels of ferroptosis-related proteins SLC7A11, GPX4, TFR1, and DMT1 by western blotting. Values are expressed as mean ± SD and were obtained from triplicate experiments. *P < 0.05 and **P < 0.01, versus the control group, ^##^ *P* < 0.01, versus 20 μg/ml CMSP.

To investigate the link between CMSP-induced mitochondrial dysfunction in SCLC cells and ferroptosis, the cells were pretreated with the mitochondria-targeted antioxidant Mito-TEMPO. The results demonstrated a significant reduction in the CMSP-induced accumulation of intracellular ROS upon Mito-TEMPO treatment, leading to a decrease in DCF fluorescence intensity (Figure 5a). Moreover, Mito-TEMPO was observed to reverse the elevated levels of CMSP-induced ROS, MDA, Fe^2+^ (Figure 5b, c, e and f), and the decreased level of GSH (Figure 5d). Additionally, the western blot results confirmed these observations by revealing the mitigating effects of Mito-TEMPO on the decreased levels of GPX4 and SLC7A11 proteins in H1688 and SW1271 cells under CMSP treatment, as well as the increased levels of DMT1 and TFR1 proteins (Figure 5g and h). These findings point to CMSP’s potential to induce ferroptosis in SCLC cells by triggering mitochondrial dysfunction.

### 3.6 Comprehensive analysis and validation of DEGs after CMSP treatment

**Figure 6:**
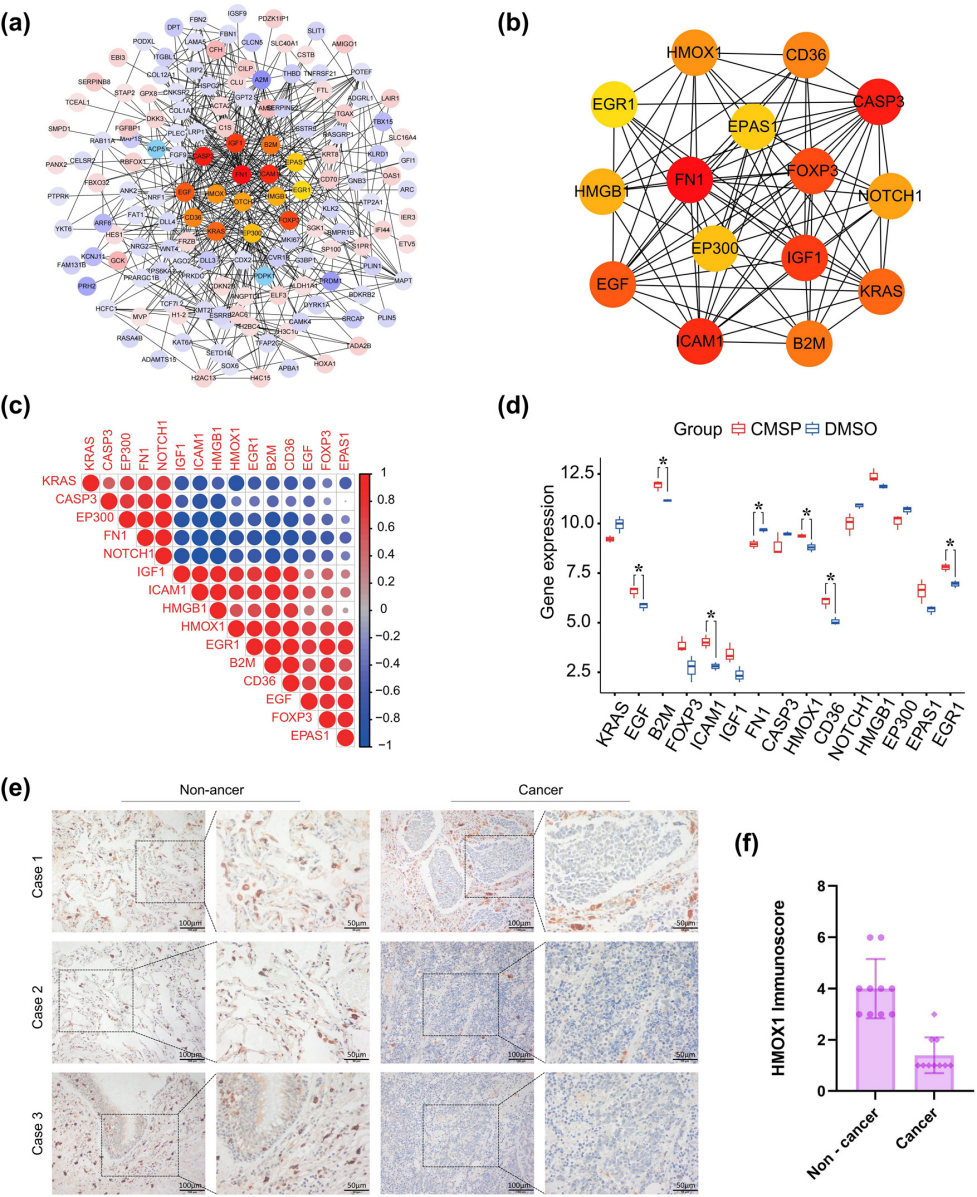
Comprehensive analysis and validation of DEGs after CMSP treatment. (a) PPI network of highly interconnected genes among DEGs of H1688 cells after CMSP treatment; (b) CytoHubba’s MCC method identifies the top 15 hub genes, with the scores being depicted in different colors: red represents a higher score and yellow represents a lower score; (c) correlation analysis of 15 hub genes; (d) mRNA expression of 15 hub genes after CMSP treatment; (e) and (f) IHC staining image and immunoscores of HMOX1 in a representative tissue section of SCLC.

**Figure 7:**
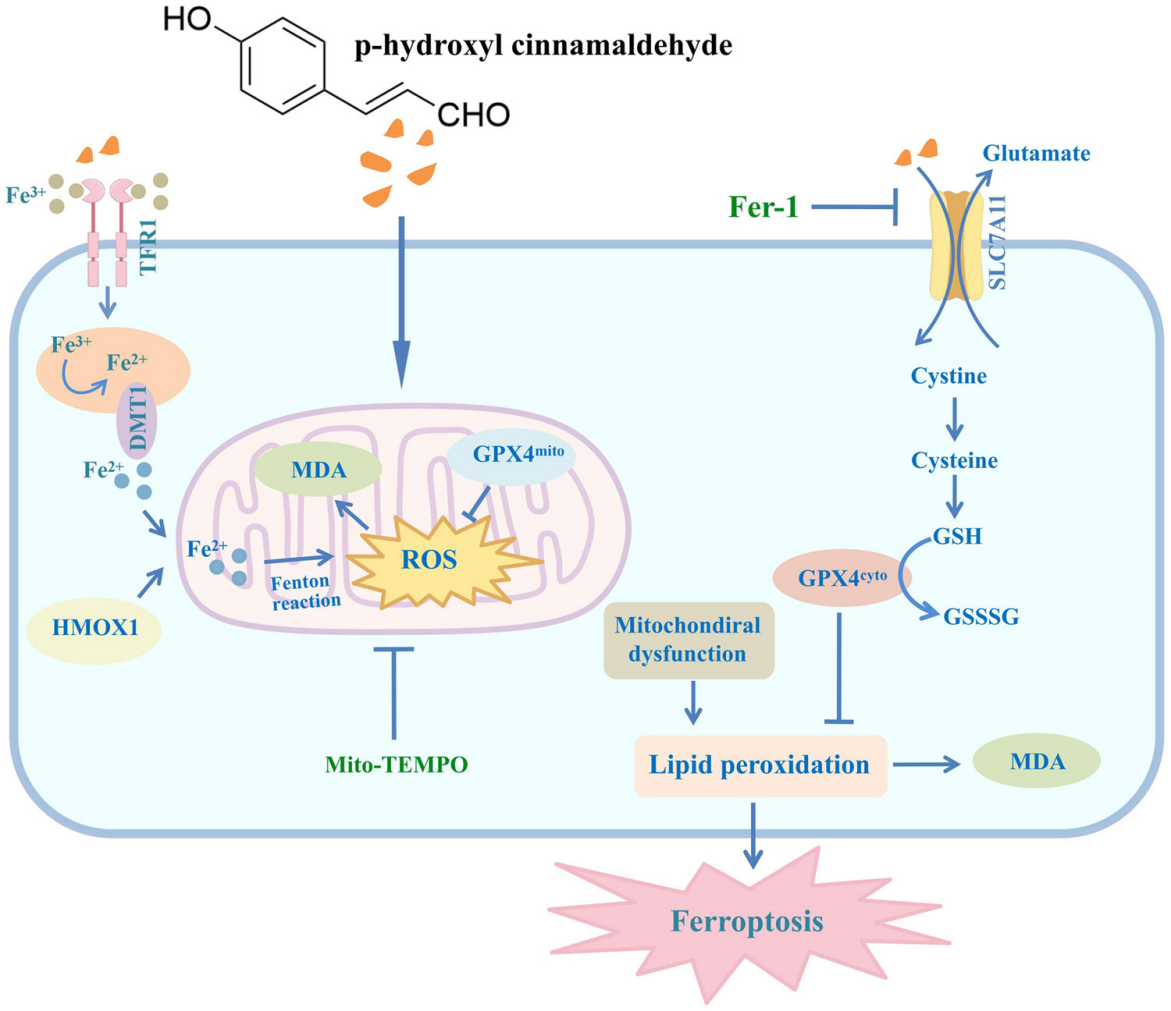
Schematic diagram of CMSP-induced pathway of cell ferroptosis. TFR1, transferrin receptor 1; DMT1, divalent metal transporter 1; ROS, reactive oxygen species; GPX4, glutathione peroxidase 4; SLC7A11, recombinant solute carrier family 7, member 11; MDA, malondialdehyde; Fer-1, ferrostatin-1; HMOX1, heme oxygenase 1.

We used STRING to construct a PPI network to study the interaction between differentially expressed genes. We visualized it using Cytoscape software (v3.9.1), calculated the Degree value, and selected the top 15 key genes, as shown in Figure 6a and b. These 15 key genes show high correlations among each other, with positive correlations between KRAS, CASP3, EP300, FN1, and NOTCH1 and positive correlations between EGF, B2M, OXP3, ICAM1, IGF1, HMOX1, and CD36. Additionally, there are negative correlations between KRAS, CASP3, EP300, FN1, NOTCH1 and EGF, B2M, OXP3, ICAM1, IGF1, HMOX1, and CD36, as shown in Figure 6c. The expression of these 15 key genes in SCLC is depicted in Figure 6d; compared to the normal group, the expression level of FN1 is significantly reduced after CMSP treatment. In contrast, the expression levels of EGF, B2M, ICAM1, HMOX1, CD36, and EGR1 are significantly increased with statistical significance (*P* < 0.05). HMOX1 has been identified as a ferroptosis protein. Validation studies revealed that HMOX1 expression scores in 10 cases of SCLC tissues (1.4 ± 0.699) were markedly lower than in adjacent tissues (4.0 ± 1.155), showing statistical significance (*t* = −7.005, *P* < 0.005, Figure 6e and f). These findings may indicate a significant role for HMOX1 in CMSP-induced ferroptosis in SCLC cells, as illustrated in Figure 7.

## 4 Discussion

Cochinchina momordica seeds, a traditional Chinese medicine, exhibit various beneficial effects including reducing swelling, dispersing masses, anti-inflammatory properties, as well as anti-cancer and immune regulatory functions. The monomeric compounds, crude extracts, and traditional Chinese medicine formulations of cochinchina momordica seeds have shown significant effectiveness in inhibiting tumor cell growth. In our previous studies, it was found that CMSP has anti-tumor effects via distinct pathways in esophageal cancer and melanoma [15,16,17]; however, its role in SCLC remains uncertain. This study provides evidence that CMSP diminishes the viability and clonogenicity of H1688 and SW1271 cells, leading to cell death induction. Bioinformatics analysis has revealed ferroptosis as a potential key pathway for CMSP-induced cell death.

Ferroptosis is a distinct form of cell death triggered by lipid peroxidation in an iron-dependent manner. This mechanism is intricately linked to the cell lifecycle and has been identified as a significant factor in a variety of tumor types, including gastric and esophageal cancer [22,23,24]. The concept of ferroptosis was initially introduced by Professor Brent R. Stockwell and his team in 2012 [25]. Subsequent studies have revealed that cancer cells exhibit a higher susceptibility to ferroptosis [26]. Consequently, the targeting of ferroptosis has emerged as a prominent focus in the realm of drug design and development.

Lipid peroxidation is a significant contributor to ferroptosis, characterized by an imbalanced redox status and significant levels of ROS within cells [27]. MDA serves as the ultimate byproduct of lipid peroxidation and is widely recognized as a key indicator for assessing the escalation of oxidative stress-induced lipid peroxidation [28]. GSH and GPX4 play pivotal roles as crucial intracellular antioxidants; a decline in their activity diminishes the antioxidant capacity of cells, potentially leading to lipid peroxidation accumulation and subsequent initiation of ferroptosis [29,30]. SLC7A11, a reverse transport protein in the body’s antioxidant system, facilitates the transport of extracellular cystine into the cell for GSH synthesis. Yang et al. validated that the inhibition of SLC7A11 can induce ferroptosis [31,32]. Consequently, GSH, SLC7A11, and GPX4 can be deemed as negative regulatory proteins for ferroptosis in tumor cells. TFR1 and DMT1 play crucial roles in the internalization of transferrin and the maintenance of iron balance within cells. Once Fe^3+^ enters the cell via TFR1, it undergoes reduction to Fe^2+^ by the ferrireductase six-transmembrane epithelial antigen of the prostate 3 (STEAP3) before being released into the cytoplasm through mediation by DMT1. The ensuing Fenton reaction triggers the production of a substantial amount of cytotoxic free radicals and ROS, consequently leading to oxidative cell death. Thus, TFR1 and DMT1 act as facilitators of iron-induced cell death [33,34,35]. In this investigation, it was observed that CMSP significantly enhances ferroptosis-associated processes, such as elevated intracellular levels of ROS, Fe^2+^, and MDA, along with reduced expressions of GSH, SLC7A11, and GPX4. Notably, CMSP also boosts the expression levels of TFR1 and DMT1 proteins. Interestingly, these effects can be counteracted by inhibitors of ferroptosis, implicating CMSP’s role in the induction of ferroptosis in SCLC cells.

The mitochondria serve as the primary sites for aerobic respiration and are responsible for generating cellular energy, and their functionality plays a direct role in influencing the cell’s viability [36]. Excessive iron load triggers lipid peroxidation, resulting in alterations in membrane fluidity and integrity, reduction in mitochondrial volume, and depletion or loss of mitochondrial cristae, ultimately leading to mitochondrial dysfunction [37]. Research indicates that mitochondrial dysfunction, which leads to the production of MtROS (mitochondrial reactive oxygen species) and depletion of ATP, is a critical factor in the initiation of ferroptosis [38,39]. You et al. demonstrated that mitochondria can mitigate oxidative stress by regulating iron metabolism to repress unstable iron production [40]. In a study by Lyamzaev et al. [41], the mitochondrial-targeted antioxidant SkQ1 was employed to prevent mitochondrial lipid peroxidation and avert ferroptosis. Our research demonstrates that CMSP induces mitochondrial dysfunction, while the mitochondrial-targeted antioxidant Mito-TEMPO mitigates this effect, highlighting the pivotal role of mitochondrial dysfunction in CMSP-induced ferroptosis.

The gene associated with ferroptosis, HMOX1, is a crucial factor in the cell’s antioxidant function, as it degrades heme to produce carbon monoxide and free iron [42]. Overactivation of HMOX1 can lead to excess Fe^2+^, triggering the Fenton reaction, resulting in the excessive generation of ROS and ultimately leading to ferroptosis [43]. Our study demonstrates an increase in cellular HMOX1 expression after CMSP intervention, with lower expression in cancer tissues compared to adjacent tissues. This leads us to speculate that HMOX1 plays a crucial role in CMSP-induced mitochondrial dysfunction promoting ferroptosis. However, the study’s limitations include the lack of *in vivo* validation of CMSP effects and the sole detection of HMOX1 expression in SCLC cancer tissues and adjacent tissues, necessitating further exploration to elucidate the effects and mechanisms of CMSP *in vivo*.

In conclusion, our research reveals that CMSP induces mitochondrial dysfunction by upregulating HMOX1 to elevate iron levels, while simultaneously inhibiting the SLC7A11/GPX4 antioxidant system to promote lipid peroxidation, ultimately leading to cell ferroptosis. This discovery implies that ferroptosis could serve as a promising target for SCLC therapy, offering significant implications for further investigations into the application of CMSP in cancer treatment.

## Supplementary Material

Supplementary material
