# Supplementary material for: CMSP exerts anti-tumor effects on small cell lung cancer cells by inducing mitochondrial dysfunction and ferroptosis
Source: Open Med (Wars). 2025 Jan 15;20(1):20241100. doi: 10.1515/med-2024-1100 (PMC11737370; doi:10.1515/med-2024-1100)
Supplement: Supplementary material [file med-2024-1100-sm.pdf]

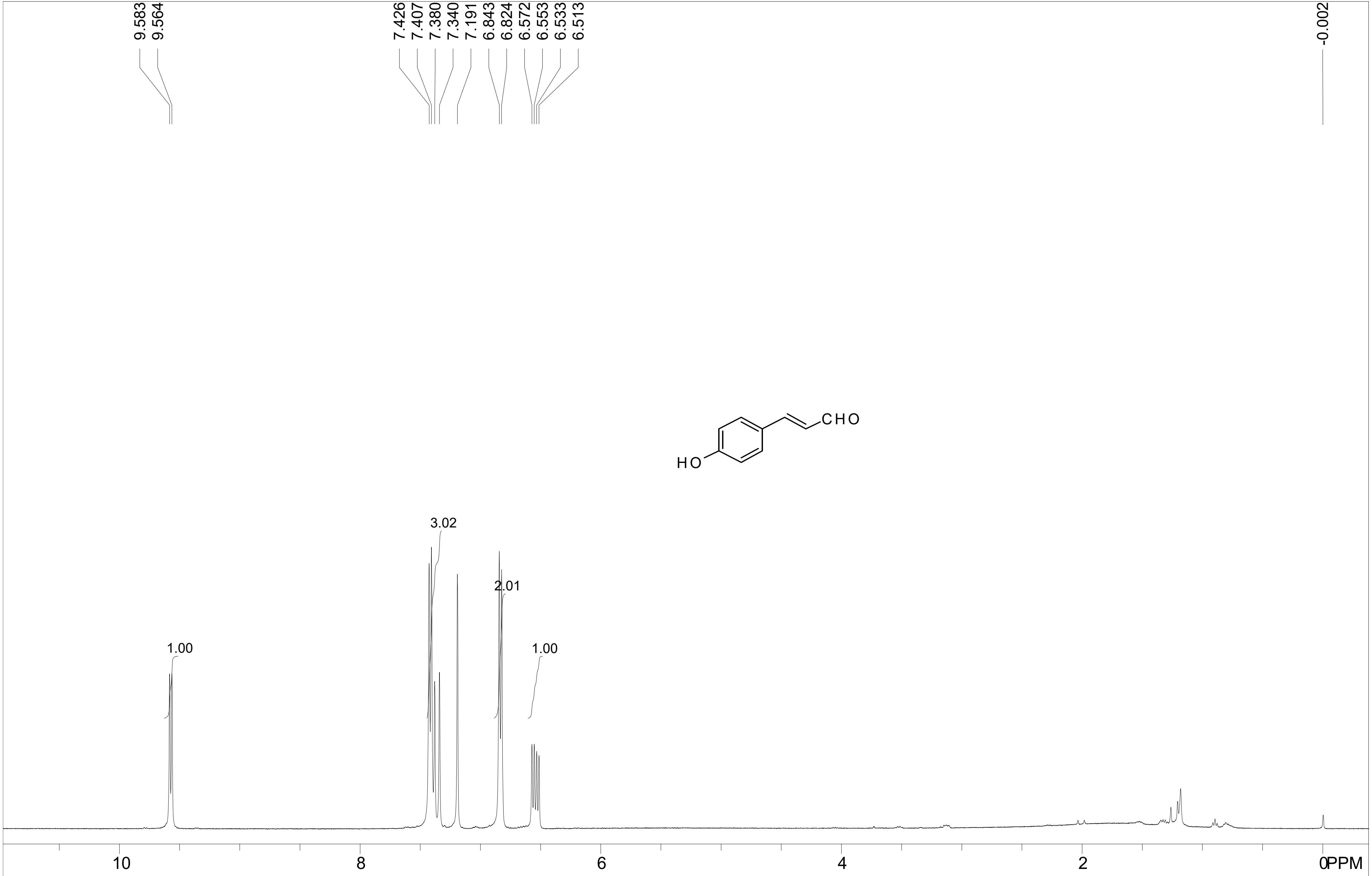

CAB, CDCl3,

F1: 400.152	F2: 1.000	SW1: 8197		OF1: 2434.3		PTS1d: 65536	
EX: zg30		PW: 9.1 usec	PD: 1.0 sec	NA: 16	LB: 0.0		Nuts - $pdata

HPLC Report

Column	: Athena, C18-WP, 50×4.6 mm,5um. HPLC001
Mobile phase	: Solvent A: H20/CH3OH/FA=90/10/0.05 Solvent B: H20/CH3OH/FA=10/90/0.05
Flow rate	: 1mL/min
Run time	: 0.4 min@ 30% B, 3.4 min gradient (30-100% B), then 0.8 min@100%B.
Temperature	: 35 ℃.
Detector	: UV
Wavelenth	: 220/254 nm
Notebook page	: LZF-N180938-106-A
Sample description	: 1.5mg/mL in CH3OH
User name	: LZF
Injection Volume	: 5 uL
Data File Name	: LZF-N180938-106-A
Method File Name	: 30-100%-5min-CH3OH.lcm

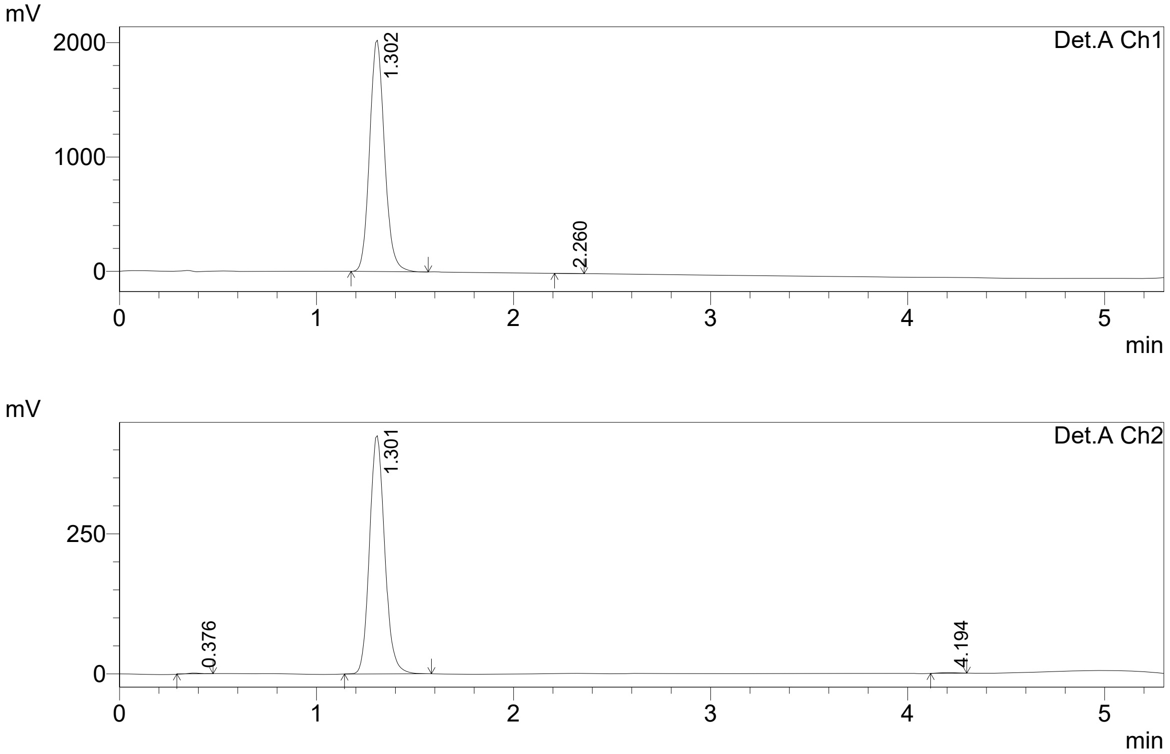

1Det.A Ch1/220nm
2Det.A Ch2/254nm

PeakTable

Detector A Ch1 220nm

Peak#	Ret. Time	Area	Area %	Theoretical Plate#
1	1.302	10599254	99.891	1186.092
2	2.260	11514	0.109	5577.908
Total		10610768	100.000	

PeakTable

Detector A Ch2 254nm

Peak#	Ret. Time	Area	Area %	Theoretical Plate#
1	0.376	6720	0.294	119.899
2	1.301	2273319	99.399	1156.000
3	4.194	7025	0.307	10987.179
Total		2287063	100.000	

LC- MS Report

LC-MS condition

Column	: Chromolith SpeedROD ,RP-18e,50*4.6mm LCMS-002
Mobile Phase	: A: H_2O(0.05%FA)
	B: CH_3CN
Temperature	: 35℃
Flow rate	: 2.5mL/min
Run time	: 0.1min@20%B,1.7min gradient(20-95% B), then0.7min@95% B,then0.4min@20% B
Injection volume	: 5 uL
Detector	: UV 205nm
Mass range	: 100-1000
Scan	: Postive/Negative
User Name	:BNN

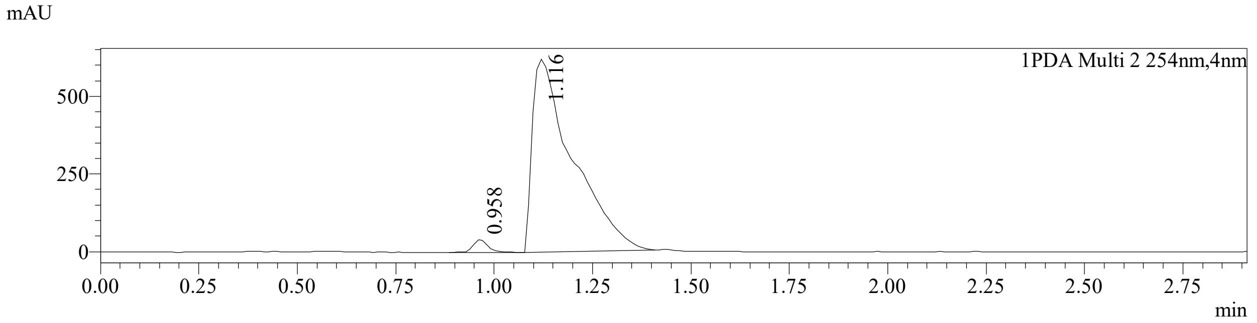

<Peak Table>

PDA Ch2 254nm

Peak#	Ret. Time	Area	Area%
1	0.958	108697	2.395
2	1.116	4429470	97.605
Total		4538167	100.000

<MS Chromatogram>

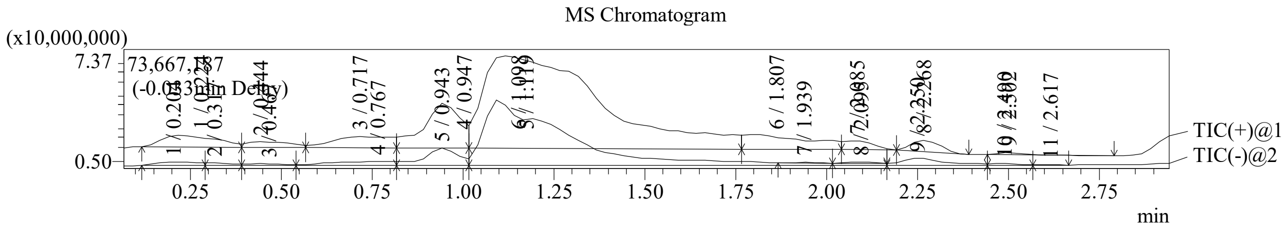

MS Spectrum

Peak#:1 R.Time:0.204(Scan#:12)
MassPeaks:548
Spectrum Mode:Averaged 0.175-0.225(10-14)
BG Mode:Calc Segment 1 - Event 2

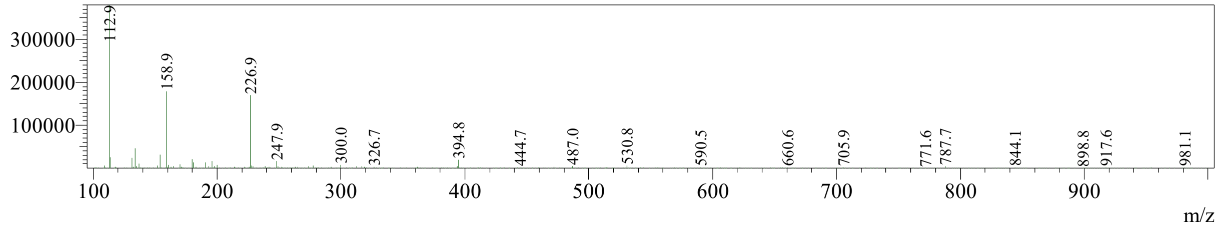

Peak#:1 R.Time:0.224(Scan#:13)
MassPeaks:572
Spectrum Mode:Averaged 0.192-0.242(11-15)
BG Mode:Calc Segment 1 - Event 1

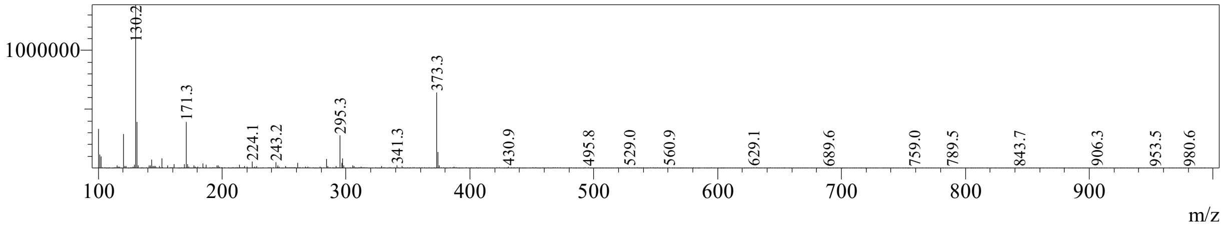

Peak#:2 R.Time:0.317(Scan#:22)
MassPeaks:424
Spectrum Mode:Averaged 0.300-0.350(20-24)
BG Mode:Calc Segment 1 - Event 2

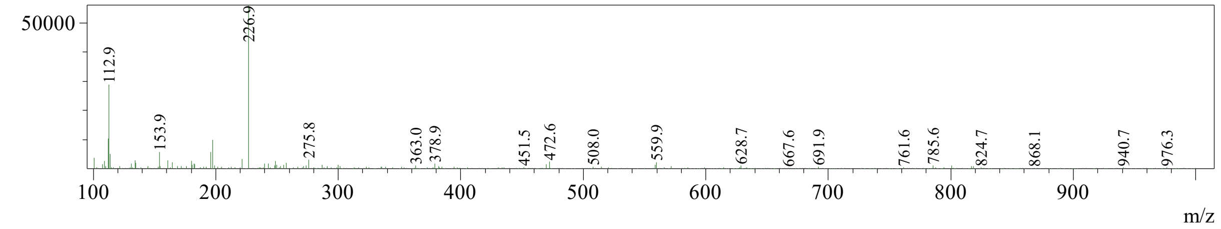

Peak#:2 R.Time:0.444(Scan#:31)
MassPeaks:495
Spectrum Mode:Averaged 0.417-0.467(29-33)
BG Mode:Calc Segment 1 - Event 1

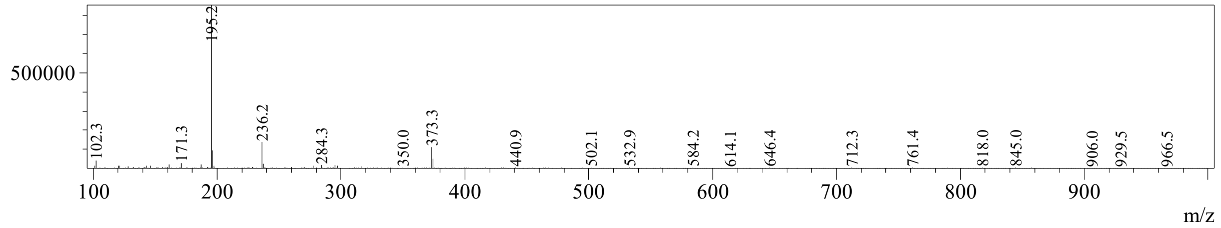

Peak#:3 R.Time:0.467(Scan#:34)
MassPeaks:476
Spectrum Mode:Averaged 0.450-0.500(32-36)
BG Mode:Calc Segment 1 - Event 2

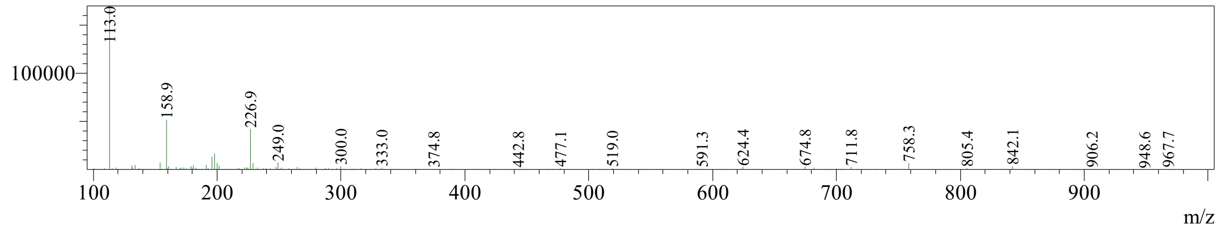

Peak#:3 R.Time:0.717(Scan#:53)
MassPeaks:497
Spectrum Mode:Averaged 0.692-0.742(51-55)
BG Mode:Calc Segment 1 - Event 1

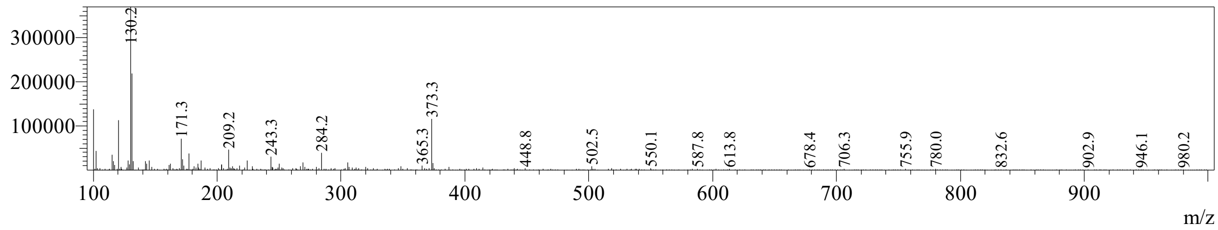

Peak#:4 R.Time:0.767(Scan#:58)
MassPeaks:473
Spectrum Mode:Averaged 0.750-0.800(56-60)
BG Mode:Calc Segment 1 - Event 2

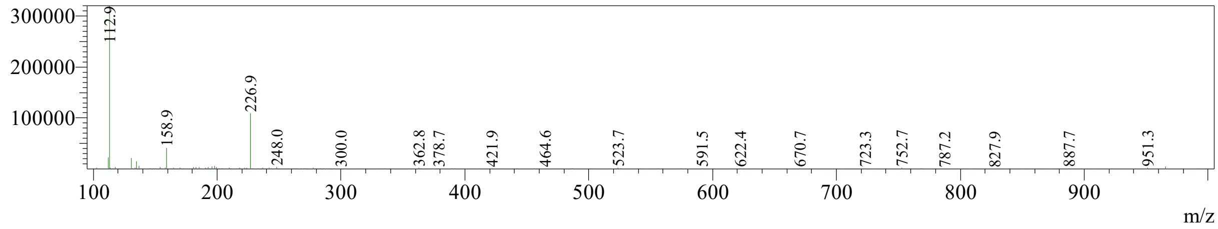

Peak#:5 R.Time:0.943(Scan#:72)
MassPeaks:472
Spectrum Mode:Averaged 0.925-0.975(70-74)
BG Mode:Calc Segment 1 - Event 2

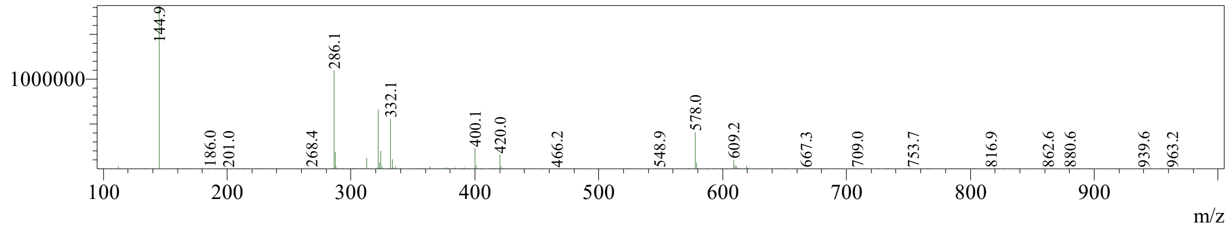

Peak#:4 R.Time:0.947(Scan#:71)
MassPeaks:503
Spectrum Mode:Averaged 0.917-0.967(69-73)
BG Mode:Calc Segment 1 - Event 1

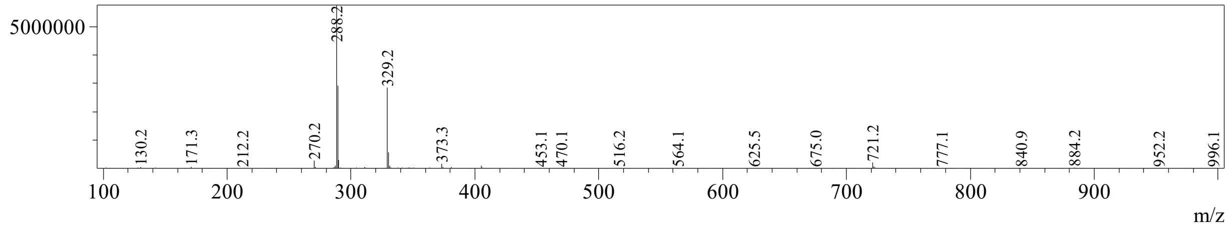

Peak#:6 R.Time:1.098(Scan#:84)
MassPeaks:601
Spectrum Mode:Averaged 1.075-1.125(82-86)
BG Mode:Calc Segment 1 - Event 2

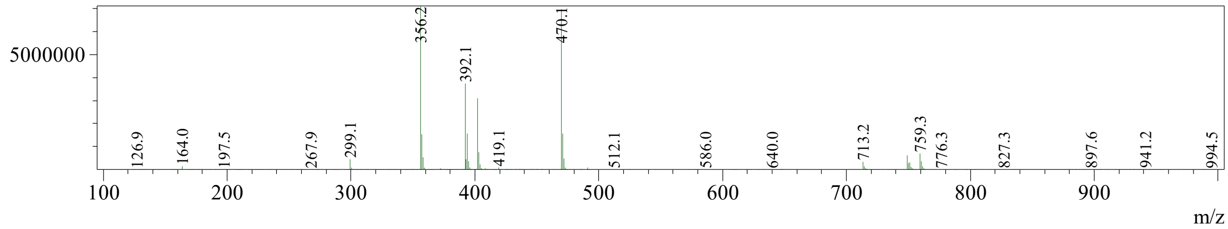

******End Of Report******
